# Superbugs online: co-production of an educational website to increase public understanding of the microbial world in, on, and around us

**DOI:** 10.3389/fmicb.2024.1340350

**Published:** 2024-02-07

**Authors:** Jon M. Tyrrell, Sarah Hatch, Melissa Flanagan, Kerry Owen, Yvonne Proctor, Catherine Stone, Geoff Fricker, Kirk Hullis, Matthias Eberl

**Affiliations:** ^1^School of Medicine, Institute of Life Science, Swansea University, Swansea, United Kingdom; ^2^Public Involvement and Engagement Team, School of Medicine, Cardiff University, Cardiff, United Kingdom; ^3^Ysgol Clywedog, Wrexham, United Kingdom; ^4^Windsor Clive Primary School, Cardiff, United Kingdom; ^5^Tredegarville Church in Wales Primary School, Cardiff, United Kingdom; ^6^Llanedeyrn Primary School, Cardiff, United Kingdom; ^7^Maxim Consulting Services, Bristol, United Kingdom; ^8^Division of Infection & Immunity, School of Medicine, Cardiff University, Cardiff, United Kingdom; ^9^Systems Immunity Research Institute, Cardiff University, Cardiff, United Kingdom

**Keywords:** public involvement and engagement, educational resources, AMR (antimicrobial resistance), infection, online learning, co-production, STEM teachers

## Abstract

Digital tools and online presence have become a cornerstone in public engagement and involvement strategy and delivery. We here describe the co-production process behind launching a new multilingual resource for schools in the United Kingdom and beyond, jointly between university scientists, engagement professionals, primary and secondary teachers, and web designers. The ‘Superbugs’ website aims at raising awareness and increasing the public understanding of the microbial world in, on, and around us—with a focus on infection, hygiene, and antimicrobial resistance—and attracted >19,000 online visitors, >33,500 page views, and > 775,000 Twitter impressions over the past 24 months. *Superbugs.online* is available in English, Welsh, Irish, and Scottish Gaelic, thus making it accessible to everyone in the United Kingdom and Ireland, regardless of the language in which they receive and deliver their science education. The website is easy to navigate and features background information, quizzes, animations, videos, illustrated stories, interactive timelines, games, and protocols for home experiments. All materials are presented in a non-prescriptive way, aimed at allowing flexibility for the materials to be adapted to the individual needs of teachers and pupils alike. Our study has led to a demonstrable impact on the co-production team and on pupils and teachers as key stakeholders, based on a comprehensive evaluation of the co-production process itself, the impact of the end product, and the creation of lasting relationships with stakeholders and co-producers, for the mutual benefit of everyone involved.

## 1 Introduction

Digital tools and online presence have become a cornerstone in public engagement and involvement strategy and delivery (Evans-Cowley and Hollander, 2010; O’Donoghue, 2020). The COVID-19 pandemic enforced an involuntary pivot to such virtual approaches—extended periods of lockdowns, closure of public spaces and schools, and social distancing meant that public engagement activities had to adapt or stand still (Cardiff University, 2023; Eberl et al., 2023). ‘Superbugs’, a research-driven initiative at Cardiff University and Swansea University, aimed at improving public understanding of the microbial world in, on, and around us (Tyrrell et al., 2022), was no different. The original goal of ‘Superbugs’ was to investigate the mechanics of how to communicate and educate on complex topics such as microbiology and antimicrobial resistance (AMR) through novel engagement strategies in public spaces (Tyrrell et al., 2022). When it became impossible to conduct in-person outreach throughout most of 2020 and 2021 as a result of COVID-19 restrictions, the aspiration shifted toward widening the scope and catchment of communicative work around research and education, condensing the enthusiasm from in-person events into an equally inspiring virtual format, and, in partnership with teachers, co-producing an interactive educational online resource.

The principle of co-production within this context may be most succinctly defined by the National Institute of Health Research (NIHR): *‘an approach in which researchers, practitioners and the public work together, sharing power and responsibility from the start to the end of the project’*, based on the assumption *‘that those affected by research are best placed to design and deliver it’* (National Institute of Health and Care Research, 2023). Central to this is the fact that the partners in the co-production process would also be the primary beneficiaries of its outcomes. In the case of Superbugs, we specifically wanted to target teachers and students of Key Stages 2 (KS2) and 3 (KS3) (school years 3–9 in England and Wales).

A co-production approach was particularly beneficial in the creation of the website as the project coincided with the launch of the new Curriculum for Wales (Welsh Government Hwb Team, 2023). As such, a key ambition of the Superbugs project was to develop educational resources that would meet the ‘four purposes’ that aim to support children and young people to be (i) *‘ambitious, capable learners, ready to learn throughout their lives’*; (ii) *‘enterprising, creative contributors, ready to play a full part in life and work’*; (iii) *‘ethical, informed citizens of Wales and the world’*; and (iv) *‘healthy, confident individuals, ready to lead fulfilling lives as valued members of society’*. Similarly, despite a primary focus on microbes, infection, and antibiotics, we wanted to ensure that our materials spanned across all six ‘areas of learning and experience’ defined as *Health and Well-being*; *Science and Technology*; *Mathematics and Numeracy*; *Expressive Arts*; *Humanities*; and *Languages, Literacy, and Communication*. The expertise and experience of co-producing teachers were expected to help ensure that our resources aligned with the content and the spirit of this new framework.

Additionally, in line with the Welsh Government’s aim to promote and facilitate the use of the Welsh language and ensure that Welsh is treated no less favourably than English (Measures of the National Assembly for Wales, 2023), we were keen to provide all Superbugs resources bilingually from the start. This was to take into account the fact that there is a free choice of Welsh- and English-medium schools in Wales, and to make ‘Superbugs’ equally available to all children in Wales independent of the language in which they receive their education. The development of inspiring educational resources around AMR has been identified as priority by others, with target audiences spanning healthcare professionals, school children, and the general public (Lecky et al., 2011; Kpokiri et al., 2021; Njeru et al., 2023). However, provision of cutting-edge materials is typically limited to infographics, bespoke learning packs and lessons plans (Lecky et al., 2011; Hayes et al., 2020), and physical or online games (Ashiru-Oredope et al., 2022; Tarín-Pelló et al., 2022; Waruingi et al., 2023). All-encompassing interactive learning resources combining different modes of delivery and aligning to the local school curriculum remain sparse, especially in minority languages such as Welsh.

The Co-production Network of Wales outlines five key values that underpin successful co-production: (i) *‘value people and build on their strengths’*; (ii) *‘develop networks that operate as silos’*; (iii) *‘focus on what matters for the people involved’*; (iv) *‘build relationships of trust and shared power’*; and (v) *‘enable people to be change makers’* (Co-production Network for Wales, 2023). These values were kept central to our project at all times. Herein, we provide an account of our co-production process, its implementation in launching a new microbiology resource for primary and secondary schools in Wales and beyond, the evaluation of both the co-production process itself for developing educational materials and the initial impact of the end product, and the creation of lasting relationships with stakeholders and co-producers, for the mutual benefit of everyone involved.

## 2 Methods

### 2.1 Pre-production preparation

Three interactive sessions by the Co-production Network for Wales^1^ for the Superbugs core members (Tyrrell, Hatch, and Eberl) and two professional web designers (Fricker, Hullis) emphasised the underlying concepts and philosophies of co-production and how to design, implement, and evaluate the process. Recruitment of co-production partners was primarily done by the existing communication network of all schools across Wales, which was compiled by the Cardiff University School of Medicine Engagement Team. While being limited to virtual meetings due to COVID-19 restrictions in place at the time (Frobel et al., 2022) and not being able to take advantage from in-person teambuilding as foundation for constructive co-production, we were able to involve teachers from the whole of Wales, thus covering a geographical area of 20,000 km^2^. These virtual meetings benefited from our extensive experience with the all-Wales ‘Life Science Challenge’ inter-school competition, which features online quiz rounds (Cardiff University, 2023), and the development and delivery of online and hybrid teaching materials for both school children and university students. We recruited 25 teachers to attend an initial introductory session, delivered over Zoom, where they were introduced to the background and aims behind the ‘Superbugs’ initiative, the idea of developing an educational tool for remote learning, and their potential role in this project. Teachers represented primary and secondary schools across Wales and covered both English- and Welsh-medium education. A total of 15 teachers expressed an interest to continue to be involved in the project and became our official co-production partners.

### 2.2 Co-production process

Once partners had been recruited, co-production unfolded through three online workshops, delivered over Microsoft Teams, and facilitated using interactive tools, such as Mentimeter,^2^ Slido,^3^ and Padlet.^4^ All co-production workshops were attended by university scientists and engagement professionals, primary and secondary school teachers, and web designers to ensure that all scientific, educational, and technical aspects of the project were represented. With the official launch of the Superbugs website^5^ in October 2021, the project entered the post-co-production phase.

### 2.3 Evaluation of processes and outputs

As with our Superbugs pop-up shop project (Tyrrell et al., 2022), a multifaceted evaluator approach was taken, which was, in part, directed by the use of the ‘Measuring What Matters’ tool, provided by Co-production Network for Wales,^6^ a practical task which allowed us to identify the important questions to ask in order to rigorously achieve our evaluation aims. As a result, it was agreed that our evaluation would consist of four distinct elements.

The *horizontal evaluation* comprised of a self-assessment by project participants combined with peer review, primarily via a self-audit at the beginning, during, and at the end of the project.

For the *participatory approach evaluation*, we collected direct feedback from primary stakeholder participants through bespoke questionnaires for teachers and school children during the ‘Piloting Draft’ element of the project.

The *impact evaluation* consisted of two parts—an evaluation of the impact upon our co-production partners with regard to their own personal knowledge of microbiology and AMR and their self-reported confidence and competence in teaching these topics.

Finally, for the *product evaluation,* we assessed the success of the Superbugs website itself, gauged through monitoring website traffic, feedback collected through questionnaires and interactive elements within the website, and anecdotal feedback.

## 3 Results and evaluation

### 3.1 Outcomes of co-production workshops

**Figure 1:**
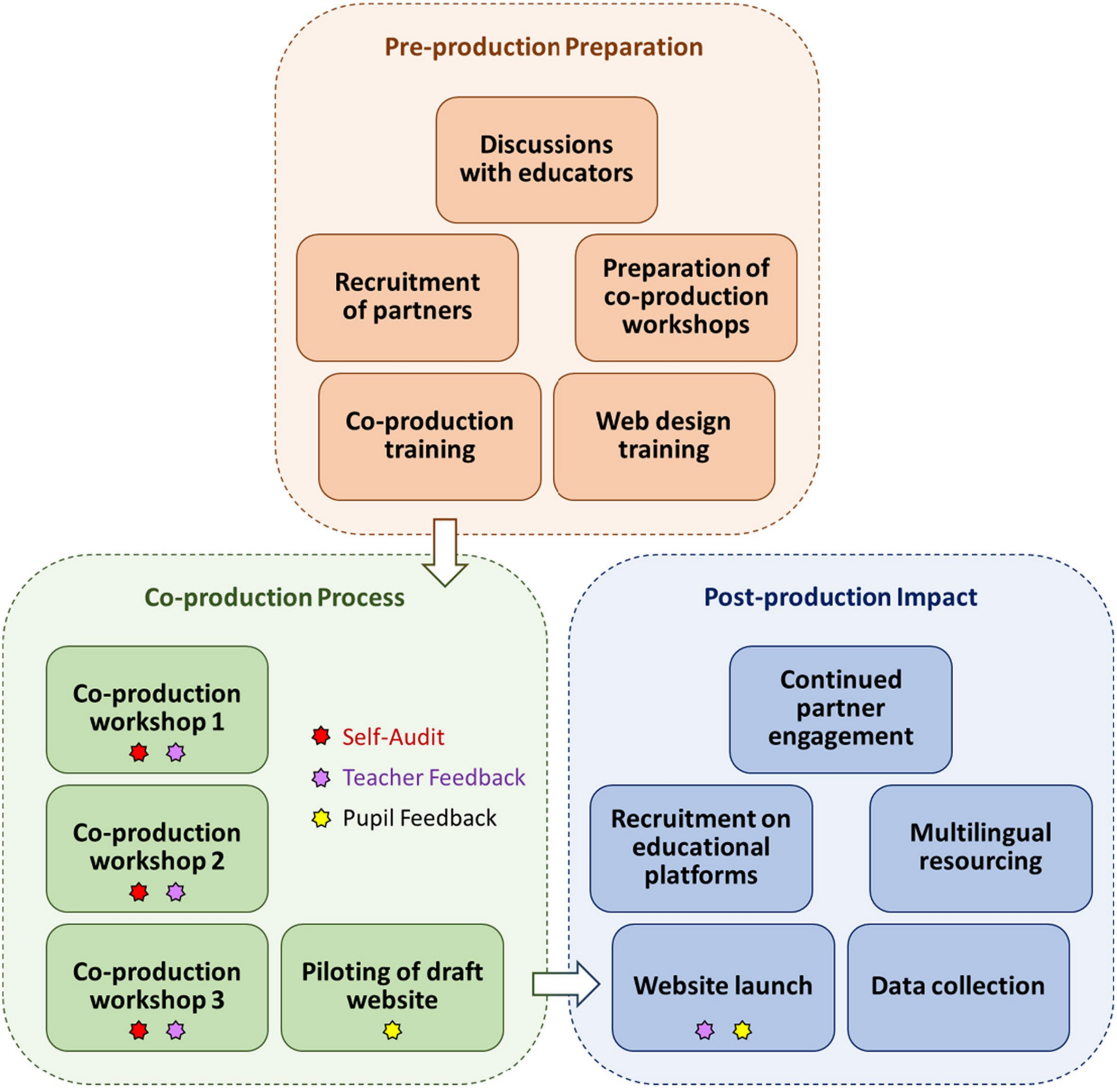
Schematic overview of the co-production project structure. Timeline: pre-production preparation, November 2020–February 2021; co-production process, March 2021–September 2021; and post-production impact, October 2021–September 2023.

**Figure 2:**
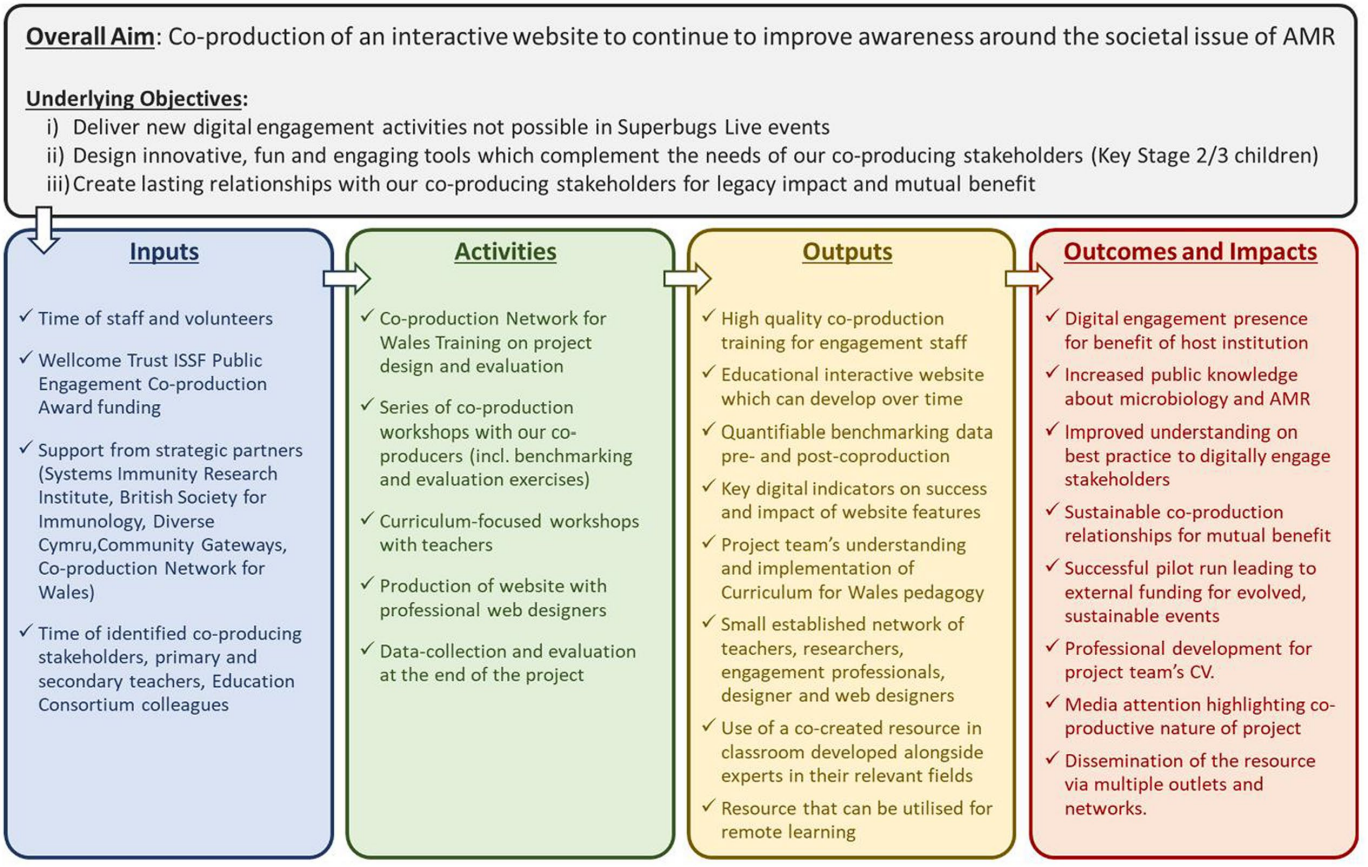
Co-produced logic model illustrating the relationship between project resources, activities, and intended impact. This is based on an initial draft that was amended and expanded during the co-production workshops.

The overall study design, including a series of co-production workshops, is visualised in Figure 1. When conceiving the project, an initial logic model was developed during the pre-production phase to define our aspirations and the processes by which we would achieve our aims and objectives. Upon recruitment of our co-production partners, the logic model evolved further to more accurately reflect the changing focus and priorities of the wider team. This revision formed a repeated exercise through the course of three co-production workshops, the final output of which is shown in Figure 2.

**Figure 3:**
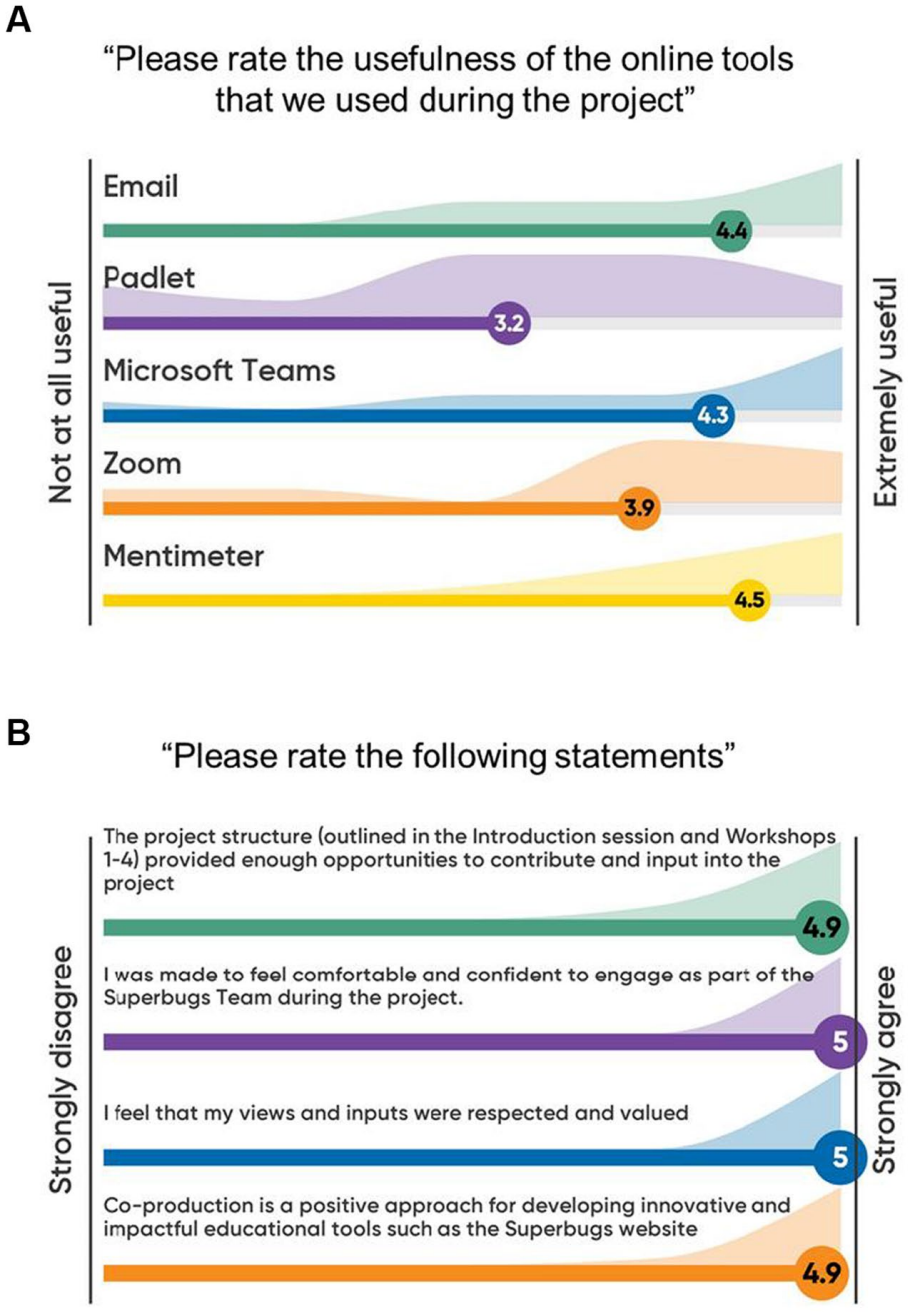
Evaluation of communication methods via electronic means and self-reflection of teachers on the co-production process. Feedback from 14 primary and secondary school teachers involved in co-producing the Superbugs website was collected during the co-production workshops using Mentimeter. Possible answers and scored ranged from 1: “*Not at all useful*” to 5: “*Extremely useful*” **(A)** and from 1: “*Strongly disagree*” to 5: “*Strongly agree*” **(B)**.

*Workshop 1* was an in-depth introduction to the project and what we were hoping to achieve. This workshop provided a useful understanding of teachers’ individual experience in designing, using, and evaluating digital teaching during the COVID-19 pandemic when schools were forced to resort to remote and hybrid learning over extended periods—a setting which was entirely novel for both teachers and pupils at the time (Table 1). In particular, insights were gained into a range of educational online platforms already employed and how best to communicate for the remainder of the project (Figure 3A). These insights formed the basis of our delivery strategy for the pilot development of *Superbugs.online*. Teachers also undertook a benchmarking exercise in the form of a simple quiz, delivered via Slido, to gauge their level of knowledge of basic microbiology.

**Table 1:** Significant outputs and discussion points from the co-production workshops.

Workshop 1: Learning from teachers’ experience in online and hybrid education.
Keep in mind how *Superbugs.online* may appear on tablets/smart phones, which is the primary method of consumption by pupils;
Link quizzes to online tools for immediate application of learning;
Provide instant feedback (pop-ups, sounds, scores etc.) on activities—pupils value being able to gauge their own progression;
Students engage well with, and learn through, competition/challenge—gamification
Embed videos and visual representation of information—avoid too much text. Ensure sections are not too long: short and sharp (approximately 10 min);
Try to make instructions for activities, either visual or oral, as opposed to text-based;
Include live broadcasts of scientists and experiments; and
Notable educational resources/tools mentioned: Google resources (Classrooms, Suite, Jamboard), Oak Academy, Kay Science, E-Bugs, Minecraft, and BBC Bitesize.
Workshop 2: A focus on the Welsh Curriculum
Consider the value of having a ‘teachers corner’ for material to be used by educators to design their own lessons and classes;
Have an unanimous desire to avoid prescript ‘lesson’ plan approach and focus on ‘simple-to-complex’ information delivery; this will allow educators to employ the material in a manner that fits their specific use;
Create interdisciplinary material spanning the six areas of learning; and
Provide elements to focus on career advice, including real life stories from actual scientists.
Workshop 3: Feedback on website elements
*Questionnaires*
students want to be able to explain WHY they have given the opinions they give in feedback;
focus on gauging ‘learning’, not just on ‘fun’; and
simplify to maximise engagement, e.g., 1–5 star rating on activities.
*Content*
Include shot quizzes at the end of each section;
Re-iteration to use videos to deliver information rather than through text;
Take the level of information back to the very basics, e.g., define what cells are, for the benefit of both pupils and teachers;
More of an interdisciplinary approach—teach microbiology through the context of other topics (maths, physics, history etc.);
Further confirmation of a preference for topic-focused material that is adaptable to a variety of teaching frameworks/scenarios. No desire for prescript lesson plans;
Highlighted the spectrum of material allowing a seamless transition from KS2 to KS3; and
Website visuals described as ‘attractive, friendly, and engaging’.

*Workshop 2* was centred around developing *Superbugs.online* in close alignment with the needs of our educational partners and was split into two parts. First, we delivered an interactive lecture introducing teachers to key concepts of microbiology, infection, and AMR. This lecture ensured that all participants were on a consistent level of knowledge for the remainder of the project and were able to engage constructively with the scientific aspects of the discussions. The second half of the workshop included discussions around the new curriculum for Wales (Welsh Government Hwb Team, 2023) to determine how our ambitions might fit into this new scheme and whether and how its confinements might affect the design and content of *Superbugs.online*. A key point raised unanimously by all teachers was the preference for a non-prescript, flexible resource that would allow educators to apply and adapt *Superbugs.online* for their own purposes. To quote our partners directly, “*Prescription takes away from being able to adapt material to* var*iety of ELOs [expected learning outcomes] across the curriculum*,” and “*having golden nuggets [of microbiology] that people can incorporate into their own teaching… would be more useful*.”

In *Workshop 3*, we began to develop the initial pages of the website to be reviewed and discussed, and co-produced feedback questionnaires that would sit across the Superbugs platform to allow for continuous monitoring of usage and feedback. Pupil feedback questions were co-produced with the help of a year 5 class at Llanedeyrn Primary School, Cardiff, and year 11 pupils at Ysgol Clywedog, Wrexham, with guidance from their teachers. Additionally, we collected valuable feedback from teachers on the pilot version of *Superbugs.online* and on how best to promote this website (Table 2).

**Table 2:** Suggestions by teachers on how best to promote our educational resources throughout schools in Wales.

Suggestion	Action taken
*“Pencils and mugs with web address into staff rooms. Teachers love pencils and mugs.”*	Purchase of Superbugs-branded merchandise such as water bottles, bookmarks, pencils, torches and rulers that are given out at live events
*“Merchandise”*	
*“Superbugs-branded chocolate biscuits”*	
*“Are there events you can attend where all schools meet?”*	Distribution of brochures at Cardiff University’s ‘Science in Health Live!’ event 2023 and at the Teacher and Career Adviser’s Conference 2023
*“At events where all schools meet.”*	
*Are there teaching meetings/conferences that we can showcase at?*	
*“Social media – Twitter.”*	Establishment of a very active Twitter profile and creation of accounts on YouTube and TikTok
*“Twitter, Facebook groups”*	
*“Facebook. Email. Hwb.”*	
*“Get in touch with* Hwb.gov.wales *and ask them to include it.”*	*Superbugs.online* now listed on the Hwb resources page of the Welsh Government and on the ‘Gaelic Education in Scotland’ page hosted by Stòrlann
*“Facebook. Email. Hwb.”*	
*“Email out to science co-ordinators”*	Targeted campaign to reach science teachers and/or science departments via direct messaging on Twitter
*“Use teachers communications network?”*	Successful contacts with science teachers across Wales and beyond via email and Twitter and establishment of long-term relationships with some of the co-production partners
*“Through the network we have developed.”*	

Feedback from 14 primary and secondary school teachers during the co-production phase of the Superbugs website.

### 3.2 Design of the Superbugs website

As a direct result of the co-production workshops, we created a website aiming to be informative, self-explanatory, and non-prescriptive and, at the same time, easy to navigate, interactive, and fun to visit (see Supplementary material). Careful consideration of software solutions and design options ensured that the final product was straight-forward to maintain and develop further by the Superbugs team, without the need for extended support by professional web designers and programmers after the initial set-up and training. *Superbugs.online* was launched bilingually in English and Welsh in October 2021 (Supplementary Figure S1, Supplementary Table S1). The ease with which the content could be hosted in different languages quickly made us realise the full potential of the project, and with the help of Stòrlann Nàiseanta na Gàidhlig, we were able to launch a Scottish Gaelic version in 2022. Grant funding from An Chomhairle um Oideachas Gaeltachta agus Gaelscolaíochta (COGG) enabled us to have an Irish version in place ready for the new school year 2023/2024. Thus, *Superbugs.online* now covers all four languages officially used for education at public and private schools across the British and Irish Isles. With a paucity of high-quality modern teaching resources in Welsh, Gaelic, and Irish (compared with English), especially in STEM subjects, this puts the Superbugs project in a unique position to fill an urgent educational need and meet the demands and ambitions from teachers and pupils (Thomas and Parry, 2021; Royal Society for Chemistry, 2023). Although the website is aligned closely to the new Curriculum for Wales, its cross-disciplinary nature and its interactive elements are relevant to teachers and pupils across the United Kingdom, Ireland, and beyond, interweaving science and health education with mathematics, humanities, languages and expressive arts, and training learners to develop numeracy, literacy, and problem-solving skills and stimulate critical thinking, creativity, planning and organising, and digital competence (Table 3).

**Table 3:** Cross-disciplinary content of the Superbugs website and alignment with the new curriculum for Wales.

**Six areas of learning and expertise**	
*Science and Technology*	Microbiology, immunology, and physiology How to use a microscope
*Health and Well-being*	Infectious diseases How the body responds to infection Difference between commensals and pathogens Hygiene, antibiotics, vaccination
*Mathematics and Numeracy*	Size of microbes in comparison to each other and other structures Exponential growth of microbes
*Languages, literacy and communication*	Resources in English, Welsh, Gaelic, and Irish Reading corner with illustrated stories Emoji quiz ‘Wordle’ and ‘Spelling Bee’ puzzles, crosswords, word searches
*Humanities*	History of scientific discoveries and medical breakthroughs Pandemic spread in an interconnected global society Collaborations with developing countries
*Expressive arts*	Drawings, colouring sheets Digital artwork, images, videos Creating bacteria-shaped balloons, play-doh models or biscuits Constructing a microbial ‘Guess Who’ game
**Skills integral to the four purposes**	
*Creativity and innovation*	Manual artwork and digital content
*Critical thinking and problem-solving*	Interactive puzzles and games
*Personal effectiveness*	Professional careers of people working ‘in science’
*Planning and organising*	Researching relevant content using a non-prescriptive educational resource
**Mandatory cross-curricular skills**	
*Literacy*	Applying reading, writing and listening skills
*Numeracy*	Understanding of mathematics applied to real-life situations
*Digital competence*	Website navigation Interactive games, animations, and simulations Creating and uploading digital content

### 3.3 Impact on co-production partners

At the end of the co-production process, the partners were asked to undergo a self-evaluation and provide feedback on individual aspects of the project. All teachers (100%) among the co-production team agreed or strongly agreed with the notion that the project structure had provided enough opportunities for them to contribute, that they had been made to feel comfortable and confident to engage, that they had felt their views and inputs were respected and valued, and that co-production was a positive approach for developing innovative and impactful educational tools (Figure 3B). Importantly, all teachers (100%) also considered that the level of their involvement had been just right for them, with nobody feeling that the level had been too little or too much (data not shown). This positive evaluation was complemented by constructive and supportive verbal feedback (Table 4), demonstrating that the co-production element had been designed optimally to benefit from involving key stakeholders in the conception, design, and implementation of the project.

**Table 4:** Self-reflection of teachers on the co-production process.

**Do you think the Superbugs project team created a positive co-production environment?**
*“Yes a fantastic and open forum to share ideas and knowledge.”*
*“Yes – I think there were constraints due to only meeting online (due to Covid) but certainly a positive environment.”*
*“Yes, time was given to discuss and share our thoughts on an equal footing with all stakeholders”*
*“Yes. Opportunities for inclusion of all. Everyone had an opportunity to share their ideas and thoughts. Everyone was listened to.”*
*“Yes, time was given to discuss and share our thoughts on an equal footing with all stakeholders”*
*“Yes i think it has been very positive. My opinions have been valued and used. All info and ways forward have been shared. It was a friendly group”*
*“Good interactive discussions in the workshops and* via *Teams”*
*“Fantastic opportunities offered through zoom meetings for all parties to contribute.”*
*“Yes, definitely – it felt like everyone was able to share their thoughts and contribute fully.”*
*“Yes, very inclusive and all ideas and opinions were valued.”*
*“Yes, excellently organised”*
*“Definitely. Everyone was able to have the opportunity to share their own ideas, thoughts, opinions to the discussions. You felt that each contribution no matter how small was always valued in the group. Expertise/knowledge/skills were valued.”*
*“It has been a very refreshing project. I have loved working with different scientists.”*
*“Yes” (3x)*
**Do you think the adopted co-production approach has been successful in achieving the intended outputs?**
*“Yes, all stakeholders were able to contribute to the final product including views from pupils within schools and clear changes have been evident throughout the coproduction.*
*“Yes—cannot add to those other comments!”*
*“Yes, the website has plenty of content that came from the initial workshops.”*
*“The end website is very different from the original idea, which has been driven by working as a co-productive team”*
*“Yes. The links with science and other curriculum areas such as Humanities has been well integrated.”*
*“I think it has been better than I expected! I think the website is just great. It is user friendly, packed with content and will be well used. All of our ideas have been incorporated in some way or another.”*
*“Yes, I think it has achieved this with regard to outputs. It’s just a pity that we have had such a high dropout rate of people throughout the workshops so we are not getting as much feedback as we would have liked.”*
*“Yes. The website is more accessible to children due to input from teachers being noted. The careful step by step approach to the development of the site gave all contributors tome to consider, experiment and respond. Timeframe suitable for teachers.”*
*“Yes, the website is fabulous and is of great value to us as teachers and to the children that we teach.”*
*“Yes, good mix of teachers and academia”*
*“very”*

Feedback from 14 primary and secondary school teachers involved in co-producing the Superbugs website.

**Figure 4:**
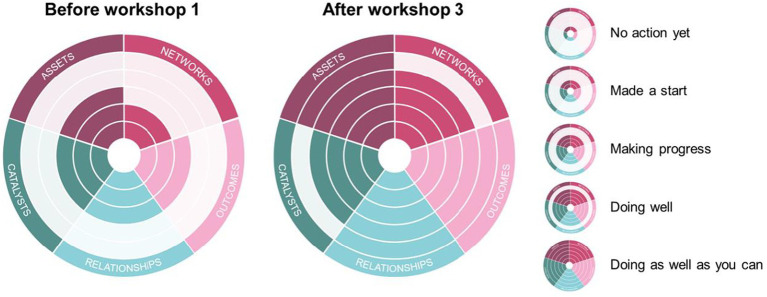
Outcomes of the self-audit at the beginning and at the end of the co-production process. Definitions: ASSETS, *“You value all participants and you build on their strengths and resources.”* NETWORKS, *“You develop networks of mutual support.”* OUTCOMES, “*You do what matters for all people involved with a focus on outcomes.”* RELATIONSHIPS, *“You build relationships of trust, reciprocity and equality by sharing power and responsibility.”* CATALYSTS, *“People are change makers, as an organisation your role is to enable this.”* Steps from the centre of the diagram to the periphery: No action yet; Made a start; Making progress; Doing well; Doing as well as you can. This tool was developed by the Co-production Network for Wales (https://info.copronet.wales/the-self-evaluation-audit-tool).

The Superbugs core team together with the web designers completed the Co-production Network for Wales’s self-audit tool before, during, and at the end of the project. At the beginning of the projects, all five categories (Assets, Networks, Outcomes, Relationships, and Catalysts) only ranked either as “*Made a start*” or “*Making progress*” (Figure 4). This initial assessment had improved considerably by the end of the project, with all five categories either ranking as “*Doing well*” or “*Doing as well as you can*,” as a testimony of the achievement and learning during the co-production phase.

### 3.4 Impact on participating teachers

It was important to the outcomes of the project that we imparted a positive impact on all co-production partners. As such, during Workshop 1, a simple benchmarking exercise was carried out to ascertain the baseline knowledge of our partners on topics around microbiology, AMR, and antibiotic stewardship. All teachers (100%) showed a basic working understanding of antibiotics, their selective toxicity against microorganisms, and some basic good stewardship practice. These competences were encouraging, as being informed to this level would enhance the intellectual level of input that could be gained throughout the co-production phase. However, many teachers suggested to have reservations in their ability to communicate/educate these topics further, with only 46% of teachers feeling confident enough to deliver teaching on AMR and microbiology. Additionally, 31% of teachers indicated at the beginning of the project that they did not feel informed enough to teach on these topics compared with 69% who answered “*A little bit*” or “*Very much*.” Importantly, this original hesitation quickly disappeared so that, by the end of the project, all teachers (100%) felt informed and confident in their abilities to design and deliver classes framed around the content of *Superbugs.online.*

### 3.5 Impact on pupils

**Figure 5:**
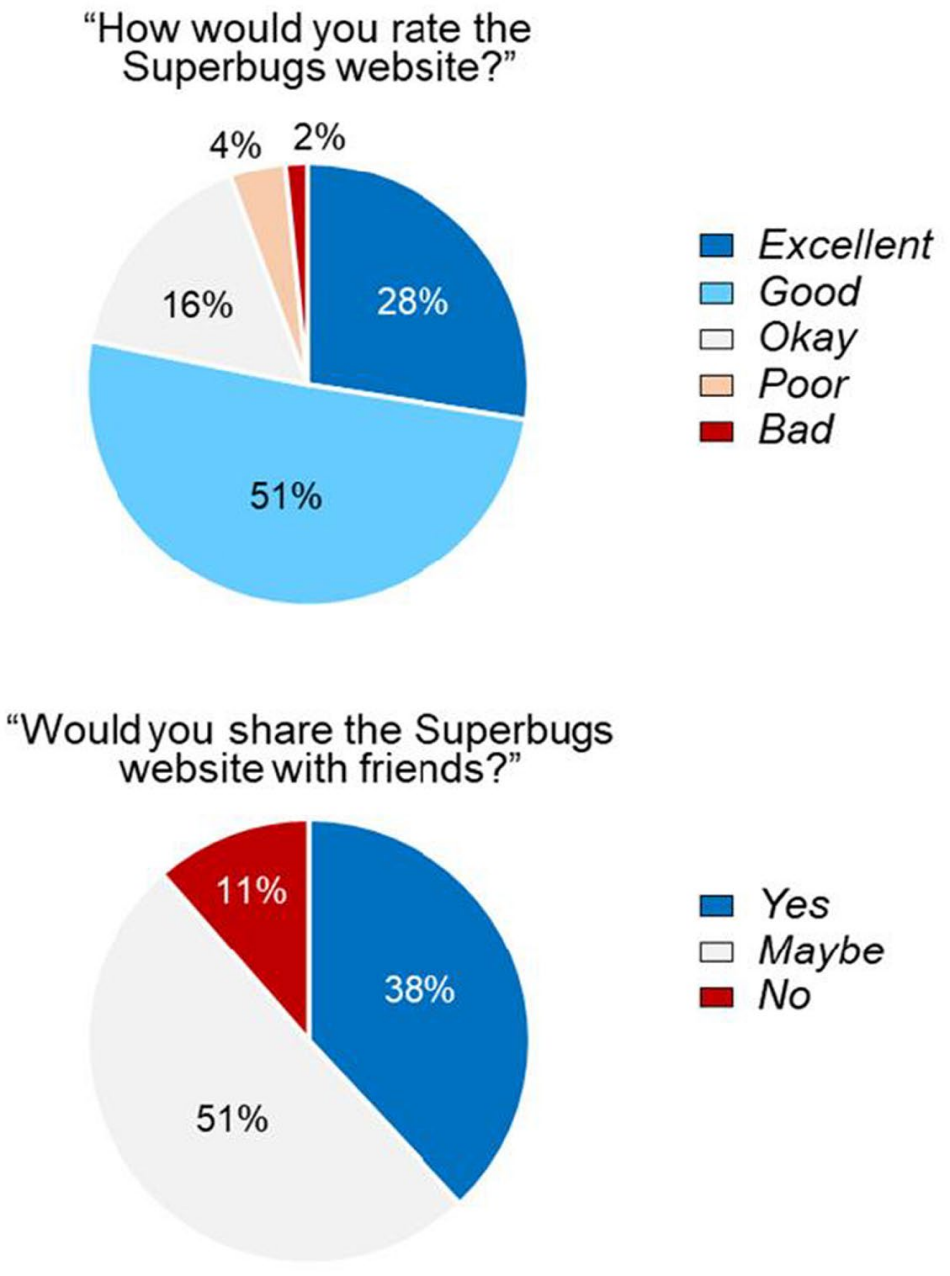
Feedback from 247 pupils attending primary and secondary schools in Wales regarding an early prototype of the Superbugs website. Pupils were from schools including Deri View Primary School (Abergavenny), Tredegarville Church in Wales Primary School (Cardiff), Ysgol Clywedog (Wrexham), and St Alban’s Roman Catholic High School (Pontypool) and completed feedback forms in July 2021, three months before the official launch of *Superbugs.online*.

During the development of *Superbugs.online*, early versions of the website were shared with the pupils of several co-production partners, and feedback from 247 pupils was received. Despite only accessing a prototype of our resources, it was reassuring that 78.1% of pupils rated the preliminary website ‘excellent’ or ‘good’ and that 38.0% would have been happy to share it with friends (compared with 11.4% who would not be happy to do so) (Figure 5). Verbal feedback emphasised the breadth of content that the students found interesting (Supplementary Table S3).

**Figure 6:**
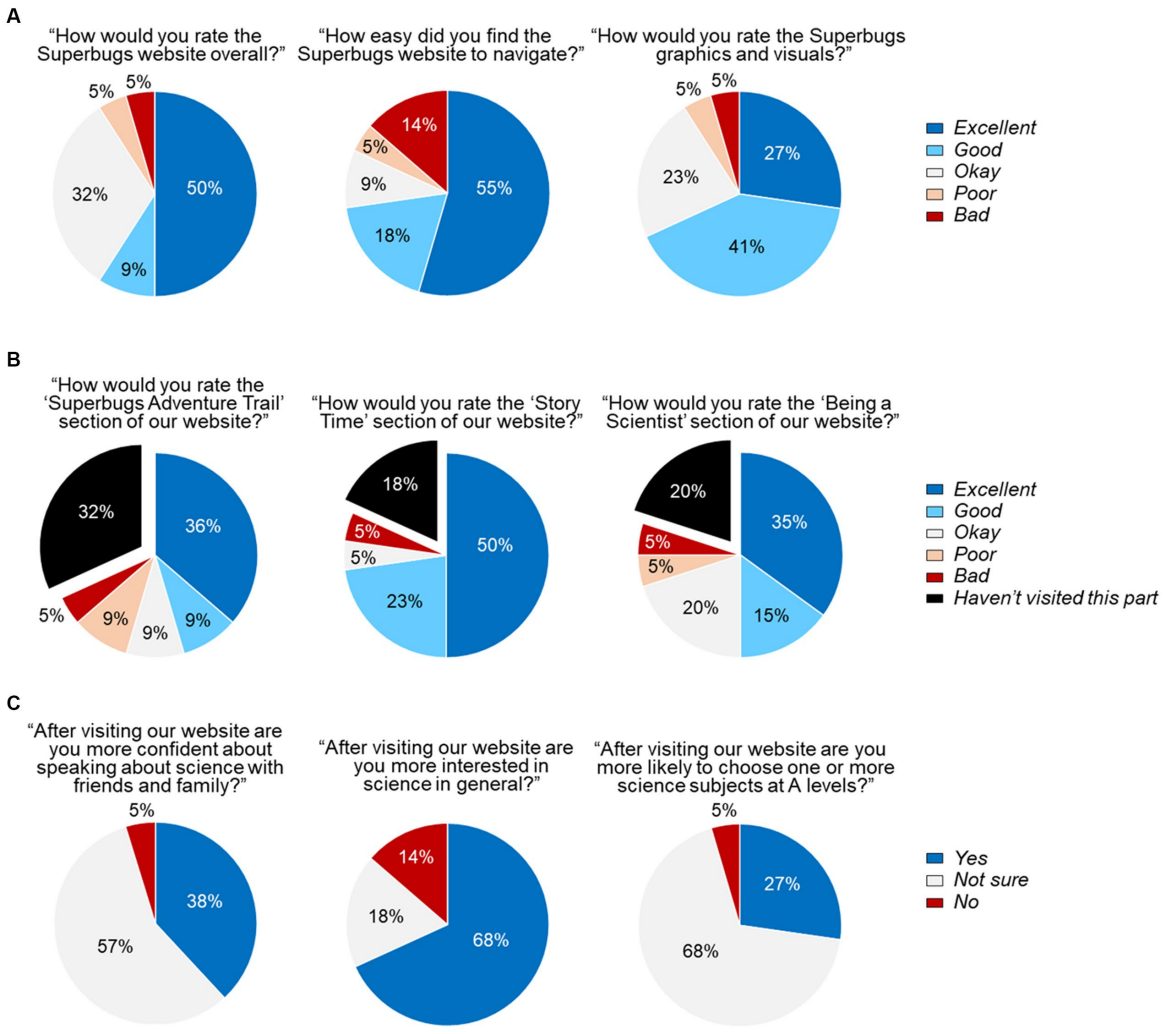
Feedback from pupils attending an English-medium primary school in Cardiff. **(A)** Overall website rating. **(B)** Ratings of major website sections. **(C)** Personal experience. Data were compiled from the answers given by 22 pupils of the same year 6 class completing the online feedback form in October 2021, shortly after the launch of *Superbugs.online*.

From the time of the official launch, student feedback was collected via feedback forms embedded in the Superbugs website. To avoid biases due to responses from individual visitors with a markedly positive or negative view of our resources, we here focused on feedback given by whole groups of students accessing the website jointly. In one particularly informative exercise at the time of the launch, all students of the same year 6 class attending an English-medium primary school in Cardiff were encouraged by their teacher to complete the online feedback form. Out of 22 submitted questionnaires, 59.1% of pupils rated *Superbugs.online* ‘excellent’ or ‘good’, 72.7% of those found it excellent or good to navigate, and 68.2% of those found the graphics and visuals excellent or good (Figure 6A).

Of the pupils who visited the corresponding sections, 66.7% of them rated the ‘Adventure Trail’, 88.9% of them rated the ‘Story Time’ section, and 62.5% of them rated the ‘Being a Scientist’ section as excellent or good. Encouragingly, only very few pupils considered *Superbugs.online* overall or aspects of it ‘poor’ or ‘bad’ (Figure 6B), and at least one of these negative responses may have been due to a mistake as it was accompanied by positive comments in the free text parts of the questionnaire (not shown). After visiting *Superbugs.online*, 38.1% of pupils felt more confident about speaking regarding science with friends and family, 68.2% of pupils were more interested in science in general, and 27.3% of pupils stated that they were more likely to choose one or more science subjects at A levels (the school leaving certificate in England and Wales). Moreover, only very few pupils gave negative responses (Figure 6C). These overall positive impressions from the questionnaires were reinforced by anecdotal comments provided by pupils about the most interesting thing they had learned and their favourite part of *Superbugs.online* (Supplementary Tables S3, S4).

## 4 Lessons learned

### 4.1 Co-production

The co-production process led to the development of lasting relationships. Several partners remained with the project in a productive and cross-disciplinary team, even after the end of the official project, and are still being consulted on many aspects of *Superbugs.online* and the wider Superbugs initiative. We very much see these partners now as a part of the core Superbugs team and, as such, no longer officially evaluate their input. Although perhaps idealistic, this observation is certainly an organic route of travel and testifies to the long-term success of our co-production. However, a major learning from this process is also that true co-production is difficult to achieve. The overall project needed to be driven by a core team, and maintaining a co-productive team with partners from external organisations turned out to require significant time and effort on behalf of key individuals to ensure that all project outputs, outcomes, benefits, and impacts were the result of true co-production.

### 4.2 Multilingual challenges

With its provision of multilingual materials, *Superbugs.online* meets a key educational need in the United Kingdom’s devolved nations (Wales, Scotland, and Northern Ireland) and the Republic of Ireland. However, accurate provision of educational content in four different languages has turned out to be more demanding than anticipated as it needs to be cross-checked by translators and education experts to make sure that the texts are both scientifically accurate and at an appropriate language level for pupils (Department of Education and Skills, 2015; Education Scotland, 2015; Thomas and Parry, 2021; Royal Society for Chemistry, 2023). It is important to note that some Superbugs content is, at present, provided in English only—e.g., certain videos, animations, and online games, where simultaneous translation is technically not possible. Ongoing work aims at replacing those English materials with multilingual alternatives and/or appropriate subtitles wherever possible. The inclusion of a glossary that lists scientific terms and explains them in simpler words for each language is planned but has not been started.

### 4.3 Promotion

Creating an educational resource is only the beginning of raising awareness and increasing understanding as it needs to be promoted heavily to make it visible and stand out against potential competitors (Hayes et al., 2020; Tarín-Pelló et al., 2022). As such, social media campaigns, direct contact with teachers, a printed brochure, and word of mouth recommendations were all important in promoting *Superbugs.online* and ensuring its relevance for science education (Supplementary Figures S2, S3). The co-production process helped considerably in this regard, allowing us to gauge the wishes and interests of teachers and pupils alike while simultaneously developing materials that directly catered for our stakeholders’ needs. As of October 2023, *Superbugs.online* was already listed as a recommended educational website by Hwb (Digital Learning for Wales, the Welsh Government’s provision of educational tools), Gaelic Education (maintained by Stòrlann Nàiseanta na Gàidhlig), and the Federation of European Microbiological Societies (FEMS), with evidence of direct web traffic referrals from each site.

### 4.4 Feedback

Collecting meaningful feedback from anonymous visitors remains a challenge. Although *Superbugs.online* features three bespoke (and co-produced) questionnaires for pupils, teachers, and other members of the public alike, uptake of this option has been very poor to date outside a supervised setting in the classroom. This is likely to be due to general survey fatigue (de Koning et al., 2021; Shiyab et al., 2023) and needs to be addressed going forward. The long-term aim is to embed more seamless evaluation strategies as part of the engaging activities. In the meantime, we will continue and intensify our collaborations with teachers and schools to learn how to improve our resources further and keep monitoring web traffic to understand which parts of *Superbugs.online* are particularly successful. To date, the web and Twitter statistics, together with direct feedback from teachers and pupils, demonstrate the effectiveness of our engagement with key stakeholders and the interest in our resources, thus directly addressing the original objectives set out in the co-production workshops.

### 4.5 Long-term investment

Developing and debugging an educational resource such as *Superbugs.online* and keeping it up to date comes with considerable expenses, even if the content itself is largely contributed free of charge. Costs for long-term website maintenance currently amount to approximately £750 per year for domain registration and Squarespace and Weglot subscriptions in addition to translation and proof-reading services. To date, our team has covered these expenses from grants related to the development and translation of *Superbugs.online;* however, sustaining a stable provision of our educational website in the long run will necessitate ongoing support.

### 4.6 Added value for in person activities

The possibility to provide online materials to complement our in-person events is an attractive option for extended engagement with the public and was successfully explored at small Superbugs workshops held during the 2023 summer holidays at Swansea Central Library (Swansea, United Kingdom) and the Broadlands Fun Day (Bridgend, United Kingdom). In addition to our ever popular activities such as a microscope station and a ‘Grow your own Microbe’ body swab station, these events also featured guided tours through *Superbugs.online* and the possibility for visitors to explore the online content at their own pace. The ‘Behind the Scenes’ blog section at present regularly contains summaries and photographs from live events, thereby increasing the audience reach and promoting in-person activities and website alike.

## 5 The future of Superbugs

The ability to develop and provide bespoke co-produced but flexible and non-prescriptive content that can be explored by learners, either in their own time or as guided by their teachers, is a unique advantage that distinguishes *Superbugs.online* from related resources such as e-Bug (Lecky et al., 2011; Hayes et al., 2020). In particular, we are not aware of any other in-depth educational materials available in minority languages such as Welsh, Gaelic, and Irish, thus filling an urgent need by teachers and school children across the United Kingdom and Ireland.

Our approach of combining public engagement and co-production with analyses into the effectiveness of the delivery and the impact lends itself to further academic training and investigation. In this regard, we have started to offer public engagement-based projects for students of the MSc Biomedical Science (Clinical Microbiology) module at Swansea University (United Kingdom). The first four projects successfully completed and addressed the development and assessment of novel in-person activities or online content, in co-production with teachers and pupils, and comprised a pre-production phase, a pilot phase in a public setting, and the final delivery at a local school (Cefn Glas Infant School, Bridgend, United Kingdom). We consider that this is a powerful way to train the next generation of scientists and equip them with a rich set of transferrable skills in science communication, education, teamwork, creativity, and evaluation.

AMR is of global concern, and improving modern science education and keeping it current and relevant is an ambition everywhere. The English content of *Superbugs.online* is naturally accessible to a worldwide audience, as reflected in the web traffic to our website from Europe, the Americas, Africa, Asia, and Australia (Supplementary Figure S4). Building on this positive momentum, we are increasingly working with international partners raising awareness in their communities to explain the underlying scientific and health principles of hygiene, infections, vaccines, and AMR, for instance, with volunteers in Dodoma, Tanzania (“Roll Back Antimicrobial resistance Initiative”), and Monrovia, Liberia (“Consummate Health & Sanitation”). We also participated in the virtual ‘Night of Science’ 2022 (bilingually in English and Ukrainian), organised by colleagues at Zaporizhzhia Polytechnic National University, Cardiff’s official partner university in Ukraine. These collaborations involve promoting and supporting local activities, participating in events and activities, and developing joint outreach programmes. Our Superbugs initiative will continue to improve microbial literacy worldwide through a growing portfolio of research-driven, innovative public engagement projects.

## Data availability statement

The original contributions presented in the study are included in the article/Supplementary material, further inquiries can be directed to the corresponding author.

## Ethics statement

This study did not classify as research involving human subjects, human material, or human data and as such did not require approval by an appropriate ethics committee. The individuals involved in this project were public involvement representatives and not participants in a research study. All individuals provided verbal consent to take part in the discussion groups.

## Author contributions

JT: Conceptualization, Data curation, Formal analysis, Funding acquisition, Investigation, Methodology, Project administration, Resources, Software, Writing – original draft, Writing – review & editing. SH: Conceptualization, Data curation, Formal analysis, Funding acquisition, Investigation, Methodology, Project administration, Resources, Writing – original draft, Writing – review & editing. MF: Conceptualization, Data curation, Investigation, Methodology, Resources, Writing – review & editing. KO: Conceptualization, Data curation, Investigation, Methodology, Resources, Writing – review & editing. YP: Conceptualization, Data curation, Investigation, Methodology, Resources, Writing – review & editing. CS: Conceptualization, Data curation, Investigation, Methodology, Resources, Writing – review & editing. GF: Conceptualization, Investigation, Methodology, Resources, Software, Writing – review & editing. KH: Conceptualization, Investigation, Methodology, Resources, Software, Writing – review & editing. ME: Conceptualization, Data curation, Formal analysis, Funding acquisition, Investigation, Methodology, Project administration, Resources, Software, Visualization, Writing – original draft, Writing – review & editing.

## Footnotes

^1^ https://www.copronet.wales

^2^ www.mentimeter.com

^3^ www.slido.com

^4^ padlet.com

^5^ www.superbugs.online

^6^ info.copronet.wales/audit-online
